# Parental perception of treatment options for mucopolysaccharidosis: a survey to bridge the gap for personalized medicine

**DOI:** 10.1186/s13023-025-03549-y

**Published:** 2025-01-24

**Authors:** Anna-Maria Wiesinger, Florian B. Lagler

**Affiliations:** 1 Institute for Inherited Metabolic Disorders, Paracelsus Medical University Salzburg, 5020 Salzburg, Austria; 2 Department of Rare Diseases, European Reference Network for Hereditary Metabolic Diseases, MetabERN, 33100 Udine, Italy

**Keywords:** Mucopolysaccharidosis, MPS, Parental perception, Attitudes, Discrete choice experiment, Personalized medicine, Individual treatment trials

## Abstract

**Background:**

Mucopolysaccharidosis (MPS) are a group of lysosomal storage diseases with substantial unmet medical needs—for both patients and caregivers. Approved therapies are limited, and the perception of investigative ones remains enigmatic.

**Method:**

Using an innovative survey concept based on the discrete choice experiment method (DEC) with neuronopathic and non-neuronopathic patient scenarios, we aimed to evaluate how parents of children with MPS perceive different approved and innovative therapies. The questionnaire was distributed via patient organizations in Germany, Switzerland, and Austria.

**Results:**

Most likely parents would choose an approach to repurposed treatments for their child (neuronopathic: 82%, 14/17 and non-neuronopathic: 94%, 16/17), followed by enzyme replacement therapy (ERT, both 88%, 15/17), hematopoietic stem cell therapy (HSCT, 70%, 12/17 and 76%, 13/17), and finally gene therapy (GT, 58%, 10/17 and 53%, 9/17). The general attitudes strongly influenced decision-making regarding treatment options. While over 80% of respondents who held a positive attitude toward ERT, HSCT, and ITTs indicated they would opt for these therapies in both neuronopathic and non-neuronopathic cases, only about half of the parents with a favorable general view of gene therapy (GT) expressed a positive perception of its likelihood as a treatment option. Furthermore, most parents found mild infections, injection site reactions (ISRs), hypertonia, and treatment-related hospitalizations acceptable and indicated patient organizations as their main source of information.

**Conclusions:**

This study provides an innovative survey method, thereby offering the rationale for a quantitative risk–benefit model and the importance of patient and caregiver-centered information dissemination, especially for innovative therapies.

**Supplementary Information:**

The online version contains supplementary material available at 10.1186/s13023-025-03549-y.

## Introduction

Mucopolysaccharidosis (MPS) encompasses a group of rare genetic disorders characterized by the accumulation of specific sugar molecules, the glycosaminoglycans (GAGs), within the lysosomes and extracellular system, leading to a range of debilitating symptoms that typically manifest in early childhood. While neuronopathic forms of MPS are associated with central nervous system (CNS) manifestations, such as cognitive impairment, both neuronopathic and non-neuronopathic subtypes exhibit somatic features that can significantly impact patients' quality of life [1]. Despite advancements in therapeutic interventions, the treatment landscape for MPS continues to present challenges, leaving patients and their families with substantial unmet medical needs. Currently available treatments, enzyme replacement therapy (ERT), and hematopoietic stem cell transplantation (HSCT) have been promising in managing some aspects of the disease, like mobility, endurance, functional outcomes, quality of life (QoL), organomegaly, respiratory and cardiac function. However, their efficacy is limited by several factors. Both therapies are unable to address all pathological and clinical features, due to insufficient penetration into target tissues, particularly bone, joints, and brain [2, 3]. Further, these treatments are associated with problems such as lack of tolerance, infusion reactions, high costs, and frequent invasive delivery. For HSCT, challenges include the impact of donor type on clinical outcomes, immunosuppression, graft rejection, and a high rate of associated morbidity and mortality [4]. In addition, to date no treatment options for eight out of the thirteen MPS types are available.

The limited efficacy of these treatments has prompted a quest for novel therapeutic approaches. Gene therapy (GT), both in vivo and ex vivo, has emerged as a potential avenue for addressing MPS at its root cause. However, with this potential for groundbreaking change comes a unique set of challenges that must be carefully navigated. Safety concerns linked to viral vectors are the biggest hurdle, especially when considering the perspectives of parents, who naturally prioritize the well-being and safety of their loved ones [5].

In the pursuit of finding effective and personalized solutions for MPS, individual treatment trials (ITTs), also known as N-of-1 trials, are a viable option. This approach not only offers an avenue for patients to access innovative therapies in the meaning of repurposing, but also serves as a platform for early, low-threshold interventions in a personalized manner [6]. Accordingly, the repurposing of immunomodulatory drugs for individual MPS patients is a rational and available treatment strategy with preclinical and clinical proof of concept in MPS [7]. Despite the promise and potential of ITTs with immunomodulatory drugs, the implementation among MPS-treating clinicians is low. Our preliminary work suggests that this is primarily due to the complex and time-consuming risk–benefit analysis physicians must perform, which acts as a barrier to their use [8].

The perspectives of parents and patients are crucial for clinical studies as well as ITTs. Therefore, it is essential to gain a comprehensive understanding of how parents perceive this readily available avenue for treatment. By bridging this knowledge gap, patients and parents can be empowered with informed choices, enabling them to actively participate in the decision-making process regarding their treatment journey. Moreover, involving patients and their parents in clinical research has been recognized as vital, not only to respect personal views but also to address ethical, financial, legal, and social implications (ELSI) that arise with each treatment approach [9]. Thus, parental perception and acceptability of treatment options are crucial to guide future research and clinical translation.

Considering the knowledge gap surrounding parental attitudes toward MPS therapies, our study aims to delve into this crucial area of research. Our quantitative questionnaire-based survey targets the perception of parents of affected MPS children in the DACH region (Germany, Austria, Switzerland). This article provides first insights into the parental perception of ITTs in comparison to other innovative treatment approaches as well as MPS gold standard therapies and reports valuable findings of parental acceptance of possible treatment-related adverse events.

We sought to better understand how different therapies are perceived and what factors influence MPS parents' final acceptance or reluctance. By examining their points of view, we aim to reveal valuable insights that can guide clinicians and researchers towards more patient-centered treatment approaches. Furthermore, this stakeholder perspective is an indispensable prerequisite for a rational benefit-risk assessment. Therefore, we aim to integrate these outcomes into the DAF tool [10] for evidence-based, high-quality ITTs in MPS.

Within this article, we introduce a pioneering quantitative survey technique based on the discrete choice experiment (DCE). This technique is recognized as a valuable method for complex decisions and has been applied in the orphan disease setting [11, 12] as well as for the evaluation of treatment perceptions [13, 14]. In our survey, we utilized this method by integrating innovative hypothetical MPS patients’ scenarios. This approach offers new, differentiated perspectives on parental attitudes toward both established and groundbreaking therapeutic approaches, including their willingness to accept potential adverse events related to treatments.

Consequently, our method allows us to provide data for a distinctive quantitative risk–benefit model to empower MPS patients and parents with new possibilities, fostering a future where personalized and innovative approaches stand at the forefront of the battle against MPS.

Thereby, embracing the potential opportunity to reshape the existing landscape of MPS treatments and to bridge the gap between scientific progress and patient-centered strategies.

## Methods

### Instrumental development

This quantitative research was carried out in accordance with the DCE approach [12] with an incorporation of novel attributes to support decision making of caregivers in the MPS field. The respective online survey was based on a validated quantitative questionnaire with revisions and a translation into German [15]. Therefore, the participation in the survey was likely limited to German-speaking parents residing in Switzerland. Additional survey items were included about the parental perception of different therapies and the personal assessment of potential treatment-related adverse events. Questions were written in a structured response, single choice format with a free text option where appropriate. The final survey contained 30 questions, categorized into five sections: (i) patient characteristics, (ii) parent characteristics, (iii) parental perception of various therapies a priori, (iv) parental perception regarding the likelihood of favoring different therapies, and (v) parental perception on the likelihood of accepting treatment-related adverse events (Suppl. info. 1).

Section “Results” evaluated the general parental attitude towards ERT, HSCT, GT, and ITTs. In contrast, Sect. “Discussion” assessed decision-making by using specific hypothetical scenarios involving both (a.) neuronopathic and (b.) non-neuronopathic patients. The following text passage gives insights into this section of the survey with ERT as an example:

- Imagine your child suffers from MPS-associated aggressive and abnormal behavior, sleep disorder, and cognitive decline with an increased need for parental care. The current symptoms are mild but clearly and substantially progressive. How likely would you choose enzyme replacement therapy for your child?
- Imagine your child suffers from MPS-associated skeletal abnormalities (dysostosis multiplex), short stature, gait disorders, and lack of energy and endurance. Your child will need a wheelchair and even more parental care. Current symptoms are mild but clearly and substantially progressive. How likely would you choose enzyme replacement therapy for your child?

The last section of the survey focused on individual assessments of the likelihood of favoring a therapy, involving the consideration of potential treatment-related adverse events weighed against specific beneficial effects. This assessment was facilitated through the presentation of scenarios featuring both (a.) neuronopathic and (b.) non-neuronopathic patients. The following patient scenario gives insights into this section of the survey with mild infections as an example here:

- Imagine your child suffers from MPS-associated aggressive and abnormal behavior, sleep disorder, and cognitive decline with an increased need for parental care. The current symptoms are mild but clearly substantially progressive. There is a 50% chance that stabilization and no further progression of the disease will be achieved. How likely would you be to take the risk of mild infections with a new treatment?
- Imagine your child suffers from MPS-associated skeletal abnormalities (dysostosis multiplex), short stature, gait disorders, and lack of energy and endurance. Your child will need a wheelchair and even more parental care. Current symptoms are mild but clearly substantially progressive. There is a 50% chance that stabilization and no further progression of the disease process will be achieved. How likely would you be to take the risk of mild infections with a new treatment?

### Survey distribution

The online questionnaire was published on the platform Survey Monkey® (San Mateo, CA, USA). A link was distributed via national patient organizations in the DACH region between April and July 2023. The developed cross-sectional survey was anonymous, and the contribution was voluntary. A cover letter explained the purpose of the study and informed consent was obtained before completion.

### Ethics approval

This questionnaire-based study was prepared and carried out by the relevant principles of the International Conference on Harmonization and Good Clinical Practice (ICH-GCP) and was approved by the Ethical Committee in Salzburg, Austria (SS22-0019-0019, Feb. 24th,2023).

### Data analysis and synthesis

Incomplete survey responses were excluded from the final sample size. For the analysis, descriptive statistics, group comparison, and bivariate correlation, via Pearson and Eta coefficient, were applied. All statistical analyses were performed using Microsoft Excel and IBM SPSS Statistics v. 27® (Chicago, IL, USA).

## Results

### Survey distribution and response

A total of 31 MPS parents from three different patient advocacy groups in the DACH region joined the online survey. Fourteen participants (45%) were excluded due to incomplete submissions. The final sample size comprised 17 complete responses—15 mothers (88%) and 2 fathers (12%). The majority of respondents lived in Germany, followed by Austria and Switzerland. The survey distribution is demonstrated in Table 1 below.

**Table 1:** Distribution of respondents (n = 31) and complete responses (n = 17) per country and patient organization. Only complete responses were included in the final analysis

Countries/patient group	Respondents N (%)	Complete response N (%)
MPS Austria	11 (35%)	6 (35%)
MPS Germany	15 (48%)	9 (53%)
MPS Switzerland	5 (16%)	2 (12%)

### Patients' characteristics

Parents of a total of 17 patients participated, with no cases of two prents from the same patient or one parent representing multiple patients. The mean average age at diagnosis was 34 months (median: 28 months, range: 10–72 months) and the current age was 17 years on average (median: 18 years, range: 4–31 years). The majority of patients had MPS Type III (n = 6), followed by MPS II, IV, VI (all n = 3), and MPS I (n = 2), no parents of patients with MPS Types VII, IX, X, or PLUS participated. Over half of the patients (59%) had severe CNS manifestations. Five patients were currently on ERT, and 5 children received neither causal nor supportive drug therapies. Antipsychotics and anticonvulsants were the most common supportive medications.

### Parental background and characteristics

All respondents had formal schooling, and nearly half had completed academic education. Most were employed, and all had health insurance, with 18% having additional private medical insurance. The majority (88%) of respondents were White, with one person identifying as Black and another as Asian. Further details characterizing the sample are outlined in Table 2 and Suppl. Info.2.

**Table 2:** Characteristics of survey respondents (MPS parents) and their children (n = 17)

*Parent of MPS patient (n, %)*	
Father	2 (12%)
Mother	15 (88%)
*Geographic ancestry (n, %)*	
Austria	6 (35%)
Switzerland	2 (12%)
Germany	9 (53%)
Employment status (n, %)	
Employed	11 (65%)
Unemployed	6 (35%)
*Healthcare coverage (n, %)*	
National health insurance	17 (100%)
Additional private medical insurance	3 (18%)
*Highest level of education (n, %)*	
Middle school	1 (6%)
High school (Matura/Abitur)	1 (6%)
Apprenticeship training	7 (41%)
Bachelor’s degree	2 (12%)
Master’s degree	4 (24%)
Graduate studies (e.g. PhD)	2 (12%)
*Patients ethnicity (n, %)*	
American Indian/Alaska Native	0
Asian	1
Black/Afro-American	1
White	15
*Patients MPS Type (n, %)*	
MPS I (Hurler/Hurler–Scheie/Scheie Syndrome)	2 (12%)
MPS II (Hunter Syndrome)	3 (18%)
MPS III (Sanfilippo Syndrome type A/B/C/D)	6 (35%)
MPS IV (Morquio Syndrome type A/B)	3 (18%)
MPS VI (Maroteaux Lamy Syndrome)	3 (18%)
MPS VII (Sly Syndrome)	0
MPS IX (Natowicz Syndrome)	0
*Patients with CNS manifestation (n, %)*	
Yes	10 (59%)
No	7 (41%)
*Patients (supportive) medication (n, %)*	
Yes	5 (29%)
No	12 (71%)

### Parents’ source of information for MPS treatment options (n, %)

The main sources of information were patient organizations (n = 15; 88%), followed by physicians (n = 13; 76%) and other MPS parents (n = 10; 59%). A small number of parents utilized the study registry clinicaltrials.gov (n = 4; 24%) and PubMed (n = 2; 12%).

### General subjective perception of benefits and disadvantages of different MPS therapies

**Fig. 1:**
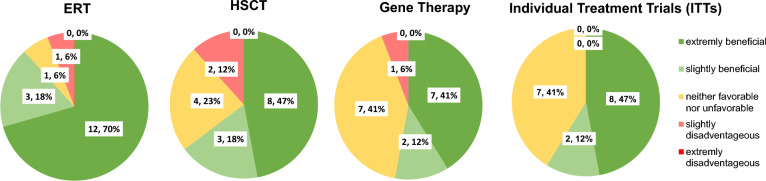
Subjective evaluation of the advantageousness of enzyme replacement therapy (ERT), hematopoietic stem cell therapy (HSCT), gene therapy (GT) and individual treatment trials (ITTs) regardless of previous experience or knowledge

Respondents demonstrated a positive attitude towards approved MPS therapies. Regardless of previous experience or knowledge, ERT received the highest rank. MPS parents perceived ERT as most beneficial, followed by HSCT (Fig. 1). Both innovative treatment approaches had a high neutral rank—41% each.

### Parental perception of the likelihood of favoring different treatment options

**Fig. 2:**
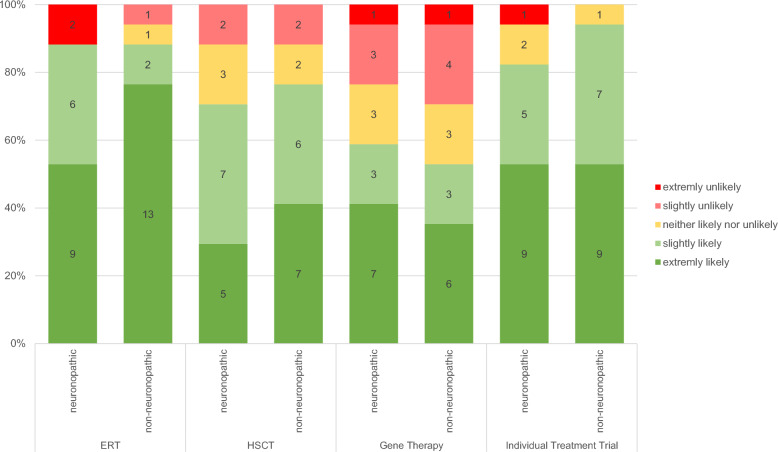
Likelihood of a decision in favor of enzyme replacement therapy (ERT), hematopoietic stem cell therapy (HSCT), gene therapy (GT), and individual treatment trials (ITTs) using neuronopathic and non-neuronopathic MPS patient scenarios

Parents were asked to rate their openness to innovative therapies in defined situations of limited efficacy of the established therapies. One scenario described a neuronopathic MPS course, while the other detailed a non-neuronopathic MPS course primarily characterized by symptoms affecting the mobility (Fig. 2). For comparative purposes, the likelihood of deciding for ERT and HSCT was assessed.

In both scenarios, all but two parents decided that it was likely or extremely likely that they would opt for ERT. Similar results apply to ITTs. A smaller number decided on HSCT and less, but still more than half of parents decided on GT. The differences between the two scenarios are minor or nonexistent, except for the percentage of extreme likelihood in favor of ERT. In the neuronopathic scenario, this percentage is much lower (35% vs. 75%), which aligns with the well-known fact that ERT does not effectively reach the CNS.

### Weighing of potential adverse events against expected benefits

**Fig. 3:**
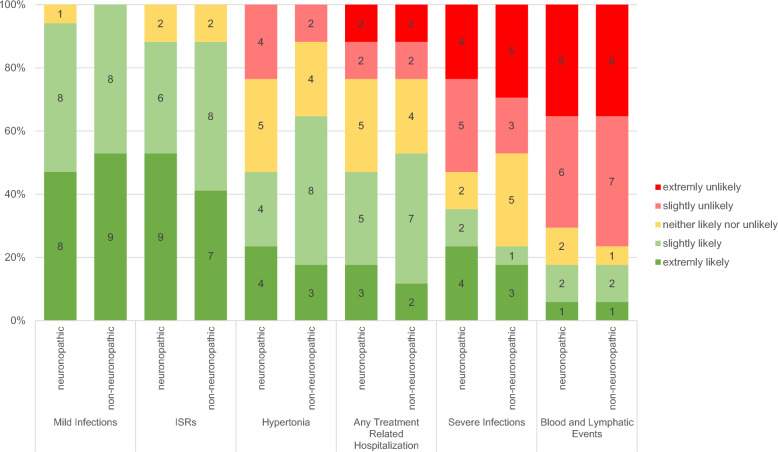
Parental acceptance of treatment-related adverse events (mild infections, severe infections, hypertonia, injection site reactions (ISRs), lymphatic events, and any treatment-related hospitalization) using neuronopathic and non-neuronopathic MPS patient scenarios

To analyze parental risk–benefit assessments regarding specific therapeutic approaches, parents were asked to evaluate which risks they would accept in exchange for a 50% chance of stabilizing either neuronopathic or non-neuronopathic disease progression. They were presented with six known risks associated with immunomodulators, which they had to weigh against the potential benefits of the therapy. The responses yielded the following ranking regarding risk tolerance, mild infections and injection site reactions (ISRs) were almost entirely tolerated as adverse effects, followed by hypertension and treatment-associated hospitalization, which approximately half of the respondents would tolerate. Severe infections and lymphatic neoplasia were acceptable to only a third or fewer of the participants. Interestingly, there was strong coherence regarding the non-acceptance ratings, showing an exactly reciprocal ranking. Specifically, lymphatic neoplasia was deemed unacceptable by 70% and 76% of respondents, while ISRs and mild infections showed no instances of non-acceptance (Fig. 3). Unlike the previous decisions, there was no difference between the neuronopathic scenario and the non-neuronopathic scenario when it came to specific risks.

**Fig. 4:**
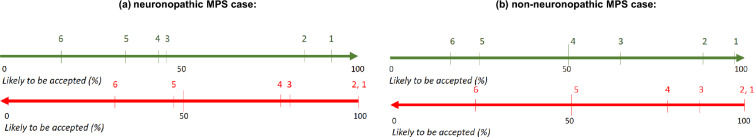
Likelihood of accepting (green) and not accepting (red) the given treatment-related adverse events in trade-off to a 50% chance for improvement in a neuronopathic MPS patient scenario (**a**) and a non-neuronopathic MPS patient scenario (**b**)—1: mild infections, 2: ISRs, 3: hypertonia, 4: any treatment-related hospitalizations, 5: severe infections, 6: lymphatic events

Using the neuronopathic MPS patient scenario, we found that the majority of parents (≥ 50%) would accept mild infections and ISRs, while using the non-neuronopathic MPS patient scenario revealed, that the vast majority of parents (≥ 50%) would accept mild infections, ISRs, and hypertonia, as well as any treatment related hospitalizations (Fig. 4).

**Fig. 5:**
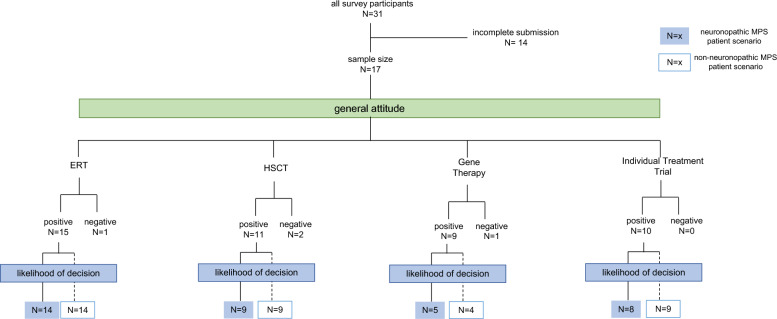
Flow chart of all participating parents in our survey—in total 17 parents provided their response on their general attitude of ERT, HSCT, gene therapy (GT), and individual treatment trials (ITTs). Survey participants with a positive attitude were further examined regarding their likelihood of favoring the respective treatment options using the neuronopathic MPS patient scenario (highlighted in blue) and the non-neuronopathic MPS patient scenario (framed in blue)

Figure 5 clearly demonstrates how the general attitude ultimately influences the decision-making process. While ≥ 80% of the respondents that showed a positive attitude towards ERT, HSCT, and ITTs would opt for the respective therapy in the specific neuronopathic and non-neuronopathic case, only half of the parents who had a positive general attitude on GT also demonstrated a positive perception on the likelihood of favoring GT.

## Discussion

Assessing the benefits and risks of therapies by patients and their relatives is critical in many medical fields, yet remains underexplored. A reliable understanding of the patient perspective is particularly critical in clinical decision-making and the development of medical innovations. While there are some insights from studies on the general attitude towards ERT, HSCT, and GT [16–20], to date, it has not been explored how the general attitude influences the actual decision for or against a specific treatment option in MPS. In this study, we have, for the first time, utilized the DCE method for this purpose and examined the relationship between the general parent assessment and the specific decision for or against established (ERT, HSCT) or innovative (ITT, GT) therapies, as well as the acceptance of relevant therapy-associated risks. A relatively high probability of choosing innovative treatment options was observed in scenarios where the effectiveness of established therapies is known to be limited. For example, in MPS IV A patients, ERT has demonstrated limited efficacy in addressing issues related to bone and joint involvement. Additionally, the tolerance for relevant adverse effects of therapy could be conclusively determined. For the first time, the utilization of the DCE approach, a highly robust method for eliciting preferences and evaluating decision-making processes in healthcare contexts, has proven to be a promising option for assessing parental decisions in the context of innovative therapies.

Our findings resonate with previous DCE studies on the general attitude of approved and innovative therapies. Llyod et al., 2017 used the DCE approach in the context of ERT to identify the relative importance of various treatment attributes in the lysosomal storage disorder Fabry disease. The outcomes of their study indicated in general a positive perception of ERT and demonstrated that besides overall survival and treatment effectiveness, also the route of administration, potential adverse effects are significant drivers of choice [20]. These results align with our findings and with the notion that approved therapies often garner more trust due to their well-known safety profiles. The general attitude among innovative therapies in previous DCE studies was consistent with our findings—mainly positive but with clear uncertainties. Specifically, the uncertainty regarding safety and long-term risks and the impact on daily life were attributes that have been closely examined within these studies on GTs [16–18]. These findings echo our outcomes regarding the general parental attitude toward GT and align with broader trends observed in the literature [21]. To our knowledge, the evaluation of the parental perception of ITTs is novel and has not been studied before.

Furthermore, and even more importantly, our project yielded a more nuanced insight into parental decision-making. A noteworthy divergence arises between parents' perceptions of innovative therapies. The general attitude of the advantageousness of GTs and ITTs was very similar with a slightly beneficial rate of 53% and 59%. However, by using specific MPS patient scenarios with a clearly defined treatment outcome, parents' likelihood of a decision in favor of ITTs significantly increased (neuronopathic 82%/non-neuronopathic 94%), while parents’ likelihood of a decision in favor of GT stagnated (neuronopathic 58%/non-neuronopathic 53%). These results signify parents' willingness to explore novel personalized interventions with potential benefits and suggest individual patient-centered approaches. Furthermore, this emphasizes the need for tailored communication strategies to ensure that MPS parents possess accurate and comprehensive information regarding all therapeutic avenues.

Interestingly, other authors have shown that families were more inclined to discontinue non-evidence-based treatments when they perceived no improvement in their child's functioning [22], emphasizing the relevance of our weighing approach. Importantly, this approach is not limited to assessing the risks associated with immunomodulatory drugs but can also be extended to treatment intended effects. Thereby facilitating a more rational decision-making process by directly comparing desired and undesired factors of therapies. We anticipate that this method holds significant relevance beyond immunomodulation, MPS, or ITTs, with implications for a wide range of clinical contexts. Considering the importance of weighing risks against benefits in clinical research, particularly in the context of clinical trials, our findings prompt critical questions regarding a meaningful and sustainable patient and parent engagement. Several studies have already indicated that besides an objective assessment of risks and benefits, also a subjective ratio of desired and undesired effects is needed [23–25]. While the first is a necessity for an informed consent, the second is crucial for the recruitment and completion of a clinical trial. Our research highlights once more the need to consider the perspective of parents in paediatric clinical research, as their insights and concerns are often overlooked but hold immense significance in shaping strategies for study outcomes, success and patient experiences [26–28].

Moreover, our survey revealed that 88% of respondents consider patient advocacy groups as their key source for treatment information and updates on MPS. This is consistent among all three countries. These findings underscore the critical role of patient organizations in ensuring that parents receive accurate, reliable, and up-to-date information about treatment options. Patient organizations can bridge the gap between medical professionals and patients. This trend might be more prominent in the DACH region compared to the US. An analysis by NORD® RARE in 2021, which highlights barriers to rare disease diagnosis, care, and treatment in the US, indicated that only 40% of rare disease patients use patient advocacy organizations as their main source of information in comparison to physicians (approx. 65%) and internet searches (approx. 75%) [29]. However, the analysis by NORD® RARE also encompasses patient organizations beyond MPS representatives, which might distort the assessment. Guffon et al. (2022) further emphasized the importance of bridging the gap between medical professionals and patients through qualitative research on the challenges, unmet needs, and expectations of MPS patients and their families in France [30].

Contrasting with the prominence of patient organizations, our study exposes an underutilization of scientific databases such as PubMed and clinicaltrials.gov. Solely 12% and 24% of the survey respondents actively use it. Interestingly, the use of these scientific databases is independent of the academic grade. Thus, the educational level might not be a proxy for parents’ capability to search and understand research articles and study protocols [15].

While our research offers important insights, the small sample size (n = 17) and skewed gender distribution (fathers: n = 2, mothers: n = 15) limits the generalizability of the findings. The high dropout rate of 45% may be caused by the survey's length, as the dropout rate increased enormously with the beginning of Section “Results” about the parental perception of therapies. In this segment, a noticeable reduction in participants occurred, declining from the initial 27–17. Thus, a selection bias in favor of participants with higher text comprehension skills cannot be excluded. However, the constantly increasing complexity ensured a valid statement from 17 participants.

One of our future aims is to capture all interested MPS parents. As the DCE approach proved to be helpful for gaining profound insights into parental perception and decision-making, the use of DCE in conjunction with interviews might be advantageous to narrow the survey's complexity. Furthermore, our study facilitates the integration of findings into a quantitative model for personalized benefit-risk assessment [31] in MPS. The occurrence probability multiplied by the personal importance allows a rational tool which can be transferred to different therapeutic contexts. We posit this should serve as a prerequisite for the accuracy in guiding clinical decision-making processes.

## Conclusion

This research sheds light on the multifaceted landscape of parental perceptions of MPS therapies by using an innovative survey concept based on the DCE approach with specific patient scenarios for a more differentiated and reliable analysis. Thereby providing the rationale for a quantitative risk–benefit model with the necessity of incorporating parents and patients voices. Acknowledging strengths and limitations, this research paves the way for a more patient-centered and informed approach to rational personalized treatment decisions.

## Supplementary Information

Additional file 1.

Additional file 2.

## Data Availability

The data that support the findings of this study are available in the supplemental information.

## References

[1] Muenzer J. Mucopolysaccharidoses. Adv Pediatr. 1986;33:269–302. 3099554

[2] Beck M. Treatment strategies for lysosomal storage disorders. Dev Med Child Neurol. 2018;60(1):13–8. 29090451 10.1111/dmcn.13600

[3] Chen HH, Sawamoto K, Mason RW, Kobayashi H, Yamaguchi S, Suzuki Y, et al. Enzyme replacement therapy for mucopolysaccharidoses; past, present, and future. J Hum Genet. 2019;64(11):1153–71. 10.1038/s10038-019-0662-9. 31455839 10.1038/s10038-019-0662-9

[4] Platt FM. Emptying the stores: lysosomal diseases and therapeutic strategies. Nat Rev Drug Discov. 2018;17(2):133–50. 10.1038/nrd.2017.214. 29147032 10.1038/nrd.2017.214

[5] Bulcha JT, Wang Y, Ma H, Tai PWL, Gao G. Viral vector platforms within the gene therapy landscape. Signal Trans Target Ther. 2021;6(1):53. 10.1038/s41392-021-00487-6 PMC7868676 33558455

[6] Schork NJ. Personalized medicine: time for one-person trials. Nature. 2015;520(7549):609–11. 25925459 10.1038/520609a

[7] Wiesinger A-M, Bigger B, Giugliani R, Scarpa M, Moser T, Lampe C, et al. The inflammation in the cytopathology of patients with mucopolysaccharidoses-immunomodulatory drugs as an approach to therapy. Front Pharmacol. 2022. 10.3389/fphar.2022.863667. 35645812 10.3389/fphar.2022.863667 PMC9136158

[8] Wiesinger AM, Strobl H, Lagler FB. Individual treatment trials-do experts know and use this option to improve the treatability of mucopolysaccharidosis? Pharmaceuticals. 2023;16(3):416. 36986515 10.3390/ph16030416 PMC10058611

[9] Hartman AL, Hechtelt Jonker A, Parisi MA, Julkowska D, Lockhart N, Isasi R. Ethical, legal, and social issues (ELSI) in rare diseases: a landscape analysis from funders. Eur J Hum Genet. 2020;28(2):174–81. 31537898 10.1038/s41431-019-0513-3 PMC6974597

[10] Wiesinger AM, Bigger B, Giugliani R, Lampe C, Scarpa M, Moser T, et al. An innovative tool for evidence-based, personalized treatment trials in mucopolysaccharidosis. Pharmaceutics. 2023;15(5):1565. 37242808 10.3390/pharmaceutics15051565 PMC10221776

[11] Toumi M, Millier A, Cristeau O, Thokagevistk-Desroziers K, Dorey J, Aballéa S. Social preferences for orphan drugs: a discrete choice experiment among the French general population. Front Med. 2020;7:323. 10.3389/fmed.2020.00323 PMC7379418 32766260

[12] López-Bastida J, Ramos-Goñi JM, Aranda-Reneo I, Taruscio D, Magrelli A, Kanavos P. Using a stated preference discrete choice experiment to assess societal value from the perspective of patients with rare diseases in Italy. Orphanet J Rare Dis. 2019;14(1):154. 31242905 10.1186/s13023-019-1126-1 PMC6595697

[13] Fischer AK, Mühlbacher AC. Patient and public acceptance of digital technologies in health care: protocol for a discrete choice experiment. JMIR Res Protoc. 2023;12:e46056. 37561559 10.2196/46056 PMC10450540

[14] Lokkerbol J, Geomini A, van Voorthuijsen J, van Straten A, Tiemens B, Smit F, et al. A discrete-choice experiment to assess treatment modality preferences of patients with depression. J Med Econ. 2019;22(2):178–86. 30501437 10.1080/13696998.2018.1555404

[15] Schwab ME, Brown JEH, Lianoglou B, Jin C, Conroy PC, Gallagher RC, et al. Fetal therapies and trials for lysosomal storage diseases: a survey of attitudes of parents and patients. Orphanet J Rare Dis. 2022;17(1):25. 35093147 10.1186/s13023-022-02178-z PMC8800365

[16] Gonzalez Sepulveda JM, Yang JC, Reed SD, Lee TH, Ng X, Stothers S, et al. Preferences for potential benefits and risks for gene therapy in the treatment of sickle cell disease. Blood Adv. 2023;7(23):7371–81. 37905989 10.1182/bloodadvances.2023009680 PMC10726244

[17] Woollacott I, Morgan G, Chowdary P, O’Hara J, Franks B, van Overbeeke E, et al. Examining patient and professional perspectives in the UK for gene therapy in haemophilia. Haemophilia. 2022;28(4):588–609. 35438818 10.1111/hae.14572 PMC9546085

[18] Witkop M, Morgan G, O’Hara J, Recht M, Buckner TW, Nugent D, et al. Patient preferences and priorities for haemophilia gene therapy in the US: a discrete choice experiment. Haemophilia. 2021;27(5):769–82. 34310811 10.1111/hae.14383 PMC9290457

[19] Sportoletti P, Laurenti L, Chiarenza A, Gaidano G, Albi E, Mauro FR, et al. Patients’ preferences for chronic lymphocytic leukemia treatment: the CHOICE study. Hematol Oncol. 2024;42(1):e3216. 37772620 10.1002/hon.3216

[20] Lloyd AJ, Gallop K, Ali S, Hughes D, MacCulloch A. Social preference weights for treatments in Fabry disease in the UK: a discrete choice experiment. Curr Med Res Opin. 2017;33(1):23–9. 27590169 10.1080/03007995.2016.1232704

[21] Bywall KS, Drevin J, Groothuis-Oudshoorn C, Veldwijk J, Nyholm D, Widner H, et al. Patients accept therapy using embryonic stem cells for Parkinson’s disease: a discrete choice experiment. BMC Med Ethics. 2023;24(1):83. 37828462 10.1186/s12910-023-00966-1 PMC10571417

[22] Bowker A, D’Angelo NM, Hicks R, Wells K. Treatments for autism: parental choices and perceptions of change. J Aut Dev Disord. 2011;41(10):1373–82. 10.1007/s10803-010-1164-y 21161676

[23] Niemeyer L, Mechler K, Buitelaar J, Durston S, Gooskens B, Oranje B, et al. “Include me if you can”-reasons for low enrollment of pediatric patients in a psychopharmacological trial. Trials. 2021;22(1):178. 33648579 10.1186/s13063-021-05119-6 PMC7923624

[24] Tsang VWL, West L, Woods C, Koh CJ, McCune S, Mullin T, et al. Role of patients and parents in pediatric drug development. Ther Innov Regul Sci. 2019;53(5):601–8. 30663334 10.1177/2168479018820875 PMC6744949

[25] Koradecka D, Pośniak M, Widerszal-Bazyl M, Augusty Nska D, Radkiewicz P. A comparative study of objective and subjective assessment of occupational risk. Int J Occup Saf Ergon. 2010;16(1):3–22. 20331915 10.1080/10803548.2010.11076826

[26] Cheng K, Mahler F, Lutsar I, Nafria Escalera B, Breitenstein S, Vassal G, et al. Clinical, methodology, and patient/carer expert advice in pediatric drug development by conect4children. Clin Transl Sci. 2023;16(3):478–88. 36510699 10.1111/cts.13459 PMC10014692

[27] Greenberg RG, Gamel B, Bloom D, Bradley J, Jafri HS, Hinton D, et al. Parents’ perceived obstacles to pediatric clinical trial participation: Findings from the clinical trials transformation initiative. Contemp Clin Trials Commun. 2018;9:33–9. 29696222 10.1016/j.conctc.2017.11.005 PMC5898566

[28] Caldwell PHY, Murphy SB, Butow PN, Craig JC. Clinical trials in children. Lancet. 2004;364:9436. 10.1016/S0140-6736(04)16942-0 15337409

[29] NORD. RARE insights report: barriers to rare disease diagnosis, care and treatment in the US. A 30-Year Comparative Analysis2020. p. 9.

[30] Guffon N, Genevaz D, Lacombe D, Le Peillet FE, Bausson P, Noel E, et al. Understanding the challenges, unmet needs, and expectations of mucopolysaccharidoses I, II and VI patients and their caregivers in France: a survey study. Orphanet J Rare Dis. 2022;17(1):448. 36564803 10.1186/s13023-022-02593-2 PMC9786416

[31] Nixon R, Dierig C, Mt-Isa S, Stöckert I, Tong T, Kuhls S, et al. A case study using the PrOACT-URL and BRAT frameworks for structured benefit risk assessment. Biom J. 2016;58(1):8–27. 25619173 10.1002/bimj.201300248

