# Supplementary material for: Parental perception of treatment options for mucopolysaccharidosis: a survey to bridge the gap for personalized medicine
Source: Orphanet J Rare Dis. 2025 Jan 24;20:36. doi: 10.1186/s13023-025-03549-y (PMC11762465; doi:10.1186/s13023-025-03549-y)
Supplement: Supplementary file 2 — Additional file 2. [file 13023_2025_3549_MOESM2_ESM.docx]

| *Parent of MPS patient (n, %), N=27* | |
| --- | --- |
| Father | 2 (7%) |
| Mother | 23 (85%) |
| Other | 2 (7%) |
| *Geographic ancestry (n, %), N=26* | |
| Austria | 11 (41%) |
| Switzerland | 2 (7%) |
| Germany | 13 (48%) |
| *Employment status (n, %), N=27* | |
| Employed | 17 (63%) |
| Unemployed | 10 (37%) |
| *Healthcare coverage (n, %), N=27* | |
| National health insurance | 27 (100%) |
| Additional private medical insurance | 6 (22%) |
| *Highest level of education (n, %), N=27* | |
| Middle school | 2 (7%) |
| High school (Matura/Abitur) | 5 (19%) |
| Apprenticeship training | 10 (37%) |
| Bachelor’s degree | 2 (7%) |
| Master’s degree | 4 (15%) |
| Graduate studies (e.g. PhD) | 4 (15%) |
| *Patients race/ ethnicity (n, %), N=27* | |
| American Indian / Alaska Native | 0 |
| Asian | 2 (7%) |
| Black/ Afro-American | 1 (4%) |
| White | 24 (89%) |
| *Patients MPS Type (n, %), N=19* | |
| MPS I (Hurler/ Hurler-Scheie/ Scheie Syndrome) | 2 (11%) |
| MPS II (Hunter Syndrome) | 4 (21%) |
| MPS III (Sanfilippo Syndrome type A/B/C/D) | 7 (37%) |
| MPS IV (Morquio Syndrome type A/B) | 3 (16%) |
| MPS VI (Maroteaux Lamy Syndrome) | 3 (16%) |
| MPS VII (Sly Syndrome) | 0 |
| MPS IX (Natowicz Syndrome) | 0 |
| *Patients with CNS manifestation (n, %), N=19* | |
| Yes | 11 (58%) |
| No | 8 (42%) |
| *Patients (supportive) medication (n, %), 19* | |
| Yes | 5 (26%) |
| No | 14 (74%) |
| *Parents’ source of information for MPS treatment options (n, %), N=22* | |
| Physicians | 13 (59%) |
| Patient organization | 22 (100%) |
| Other parents of MPS patients | 10 (45%) |
| PubMed | 2 (9%) |
| clinicaltrials.gov | 4 (18%) |
| *Parents’ subjective evaluation of the advantageousness of approved MPS therapies (n, %), N=19* | |
| Significant improvement | 5 (26%) |
| Slight improvement | 4 (21%) |
| Neither improvement nor | 4 (21%) |
| Slight deterioration | 3 (16%) |
| Significant deterioration | 3 (16%) |

**Suppl. Info. Table:** Complete sample size characteristics
